# Staphylococcal Phage in Combination with *Staphylococcus epidermidis* as a Potential Treatment for *Staphylococcus aureus*-Associated Atopic Dermatitis and Suppressor of Phage-Resistant Mutants

**DOI:** 10.3390/v13010007

**Published:** 2020-12-22

**Authors:** Yuzuki Shimamori, Shoichi Mitsunaka, Hirotaka Yamashita, Tohru Suzuki, Tomoe Kitao, Tomoko Kubori, Hiroki Nagai, Shigeki Takeda, Hiroki Ando

**Affiliations:** 1 Department of Microbiology, Graduate School of Medicine, Gifu University, 1-1 Yanagido, Gifu 501-1194, Japan; phage.darakeno.shima@gmail.com (Y.S.); s_mitsu@gifu-u.ac.jp (S.M.); kitao@gifu-u.ac.jp (T.K.); tkubori@gifu-u.ac.jp (T.K.); hnagai@gifu-u.ac.jp (H.N.); 2 Graduate School of Science and Technology, Division of Molecular Science, Gunma University, 1-5-1 Tenjin-cho, Kiryu, Gunma 376-8515, Japan; 3 Laboratory of Phage Biologics, Graduate School of Medicine, Gifu University, 1-1 Yanagido, Gifu 501-1194, Japan; 4 Department of Pharmacology, Graduate School of Medicine, University of the Ryukyus, 207 Uehara, Nishihara, Okinawa 903-0215, Japan; hyamash@med.u-ryukyu.ac.jp; 5 Genome Microbiology Laboratory, Faculty of Applied Biological Sciences, Gifu University, 1-1 Yanagido, Gifu 501-1193, Japan; suzuki@gifu-u.ac.jp; 6 G-CHAIN, Gifu University, 1-1 Yanagido, Gifu 501-1194, Japan

**Keywords:** bacteriophage, phage therapy, atopic dermatitis, *Staphylococcus aureus*, *Staphylococcus epidermidis*

## Abstract

Atopic dermatitis is accompanied by the abnormal overgrowth of *Staphylococcus aureus*, a common cause of skin infections and an opportunistic pathogen. Although administration of antibiotics is effective against *S. aureus*, the resulting reduction in healthy microbiota and the emergence of drug-resistant bacteria are of concern. We propose that phage therapy can be an effective strategy to treat atopic dermatitis without perturbing the microbiota structure. In this study, we examined whether the *S. aureus* phage SaGU1 could be a tool to counteract the atopic exacerbation induced by *S. aureus* using an atopic mouse model. Administration of SaGU1 to the back skin of mice reduced both *S. aureus* counts and the disease exacerbation caused by *S. aureus*. Furthermore, the *S. aureus*-mediated exacerbation of atopic dermatitis with respect to IgE plasma concentration and histopathological findings was ameliorated by the application of SaGU1. We also found that *Staphylococcus epidermidis*, a typical epidermal symbiont in healthy skin, significantly attenuated the emergence of SaGU1-resistant *S. aureus* under co-culture with *S. aureus* and *S. epidermidis* in liquid culture infection experiments. Our results suggest that phage therapy using SaGU1 could be a promising clinical treatment for atopic dermatitis.

## 1. Introduction

Atopic dermatitis (AD) is a common inflammatory skin disease that is known to be accompanied by abnormal overgrowth of *Staphylococcus aureus* around affected areas [1]. In general, antibiotics are used to treat severe AD; however, they clear both pathogenic and beneficial bacteria. In addition, antibiotic use accelerates the emergence of drug-resistant bacteria, further reducing treatment effectiveness [2]. Therefore, an ideal AD treatment strategy would maintain or restore the skin microbiota structure while specifically eliminating *S. aureus*. Accordingly, trials have shown that transplantation of *Staphylococcus epidermidis*—a typical epidermal symbiont in healthy skin—could be an effective strategy to improve and recover the skin microbiota of AD patients [3].

Phage therapy, a method of treating bacterial infectious diseases using the lytic activity of bacteriophages (hereafter, phages), which are viruses that infect bacteria, is a fascinating strategy for editing microbiota. Phages exhibit host specificity and infect a limited type of bacteria [4]. This specificity makes phage therapy a promising strategy for the selective elimination of pathogenic bacteria in environments with the co-existence of several types of bacteria. Phage therapy has also been applied to intestinal bacterial microbiota [5,6,7] and the treatment of burns, foot ulcer, and chronic otitis. [8,9,10].

In this study, using a mouse model, we examined the efficacy of the *S. aureus* phage in treating AD. We recently isolated the phage SaGU1 from a sewage treatment plant in Japan [11]. SaGU1 belongs to the *Myoviridae* family and infects *S. aureus* strains previously isolated from the skin of patients with AD. The SaGU1 genome carries neither toxic nor antibiotic resistance genes. SaGU1 can infect a broad range of *S. aureus* from AD patients, whereas it does not kill strains of the symbiotic bacterium *S. epidermidis*. In addition to SaGU1, we also investigated the application of *S. epidermidis* in combination with SaGU1 for inhibiting the emergence of phage-resistant *S. aureus*. Overall, our findings highlight that SaGU1 possesses sufficient efficacy against AD and that the combinatorial use of probiotics and phages can be effective in the treatment of *S. aureus*-associated AD.

## 2. Materials and Methods

### 2.1. Strains and Bacterial Growth Conditions

In our recent study, a bacteriophage, SaGU1, was isolated from sewage in Gifu, Japan [11]. Phage SaGU1 has a broad host range and affects *S. aureus* clinical isolates from AD patients but does not kill *S. epidermidis*, a symbiotic bacterium in skin microbiota [11]. In this study, *S. aureus* SA-1 from AD patients and *S. epidermidis* SE-4 from healthy volunteers in our *Staphylococcus* collection were used.

For in vitro experiments, all bacteria were grown in lysogeny broth (LB) (BD, Tokyo, Japan) at 37 °C with agitation.

### 2.2. In Vitro Bacterial Killing Assays

Overnight cultures of clinical isolates were diluted to approximately 10^4^ colony forming units (CFU)/mL. Bacterial cultures were mixed with phage lysates (multiplicity of infection (MOI) = 1, 10, 100, or 1000) and incubated. At each time point, bacteria were collected, washed twice with LB [12], serially diluted, plated onto LB plates, and incubated at 37 °C. Colonies were counted to calculate CFU/mL.

### 2.3. Co-Cultivation of S. aureus and S. epidermidis

Approximately 10^4^ CFU/mL of *S. aureus* SA-1 and 10^4^, 10^5^, or 10^6^ CFU/mL of *S. epidermidis* SE-4 were mixed and incubated at 37 °C with agitation. If needed, bacterial cultures were mixed with phage lysates (MOI = 1, 10, 100, or 1000). At each time point, bacteria were collected, washed twice with LB, and serially diluted. A total of 100 µL of each diluted sample was spread on an LB plate with or without 2.0 µg/mL levofloxacin (LVFX) (Tokyo Chemical Industry, Tokyo, Japan). In our recent study, we found that *S. aureus* SA-1 and *S. epidermidis* SE-4 are resistant and susceptible, respectively, to LVFX [11]; thus, we can separate and count both types of bacteria to calculate CFU/mL by using LB plates with or without LVFX.

### 2.4. Plaque Formation Assays

We mixed 200 µL of bacterial overnight cultures and 3 mL of LB soft agar and poured the mixture onto LB plates. Ten-fold serially diluted SaGU1 lysate was spotted onto LB soft agar and incubated at 37 °C. The efficiency of plating (EOP) was determined using the following equation:

EOP = PFU on target bacteria/PFU on host bacteria

### 2.5. Adsorption Assays

We mixed 150 µL of ~10^8^ CFU/mL SA-1 or the mutant SA-1 and 150 µL of ~10^4^ PFU/mL SaGU1 lysate and incubated at RT for 1 h. After centrifugation at 13,000*× g* for 1 min, we added drops of chloroform to 200 µL supernatants to kill the cells and prevent the production of progeny phages. After centrifugation at 13,000*× g* for 1 min, 100 µL of supernatants, 200 µL of bacterial overnight culture, and 3 mL of LB soft agar were mixed and poured onto LB plates. After incubation, phage plaques were counted, and adsorption efficiencies were calculated according to the following equation:

$$Adsorption\;efficiency\;\left(\%\right)=\;[\;1-\left(\frac{PFU\;of\;unadsorbed\;phage}{original\;PFU\;in\;the\;host\;bacterium\;and\;phage\;mixture}\right)]\times 100$$

### 2.6. Animal Test

Six-week-old female BALB/c mice were purchased from the Charles River, Japan. They were maintained at constant room temperature (23 °C ± 2 °C) with free access to water and food under a 12:12 light:dark cycle and a specific pathogen-free (SPF) environment. All animal experiments were performed in accordance with the Animal Care and Use Committee of Gifu University (approval #2019-202). A total of 84 mice were randomly divided into six duplicate groups (n = 7 per group; group A for CFU, and group B for IgE ELISA and skin histology analysis). AD-like skin lesions were induced in mice using 2,4-dinitrochlorobenzene (DNCB) (Kanto Chemical, Tokyo, Japan) [13,14]. DNCB was dissolved in an appropriate solvent (acetone:olive oil = 3:1). Mice were sensitized by applying 100 µL of 1% DNCB or solvent to their shaved back skin from day −13 to day −9 every other day, and by applying 100 µL of 0.2% DNCB or solvent to the back skin from day −7 to day −1 every other day. After sensitization, the mice were challenged on day 0 by applying 10^5^ CFU of *S. aureus* SA-1 on their back skin. The control mice were painted with the solvent alone. One hour later, 10^7^ CFU of *S. epidermidis* SE-4 and/or phage SaGU1 (10^8^ PFU, MOI = 1000) were applied.

On day 1 (24 h after treatment with *S. epidermidis* and/or phage SaGU1), the back skin of the animals in group A was cut and collected. After weighing, the skin samples were washed twice with phosphate-buffered saline (PBS), re-suspended in PBS, and serially diluted. A total of 100 µL of each diluted sample was spread on an LB plate with or without LVFX as described above and incubated at 37 °C, and bacterial colonies were counted to calculate CFU/mL.

Blood sampling was performed on day 3 from group B using heparin-treated capillaries. The amount of IgE in the plasma sample was determined using an ELISA kit according to the manufacturer’s instructions (mouse IgE EIA kit, Yamasa, Tokyo, Japan). On day 5, the mice in group B were euthanized and used for skin histology analysis. The pieces of the back skin of the mice were fixed with 10% formalin (FUJIFILM Wako Pure Chemical, Osaka, Japan). Paraffin-embedded blocks were prepared, and hematoxylin-eosin staining was performed at the Tohkai Cytopathology Institute (Gifu, Japan).

### 2.7. Statistical Analysis

Statistical analyses were performed using Prism 8.4.3 (471) (GraphPad Software, San Diego, CA, USA). The data are presented as the mean ± standard deviation (SD) of at least three independent experiments. Student’s *t*-test was used for statistical analysis to determine significance.

## 3. Results

### 3.1. Growth Inhibition of S. aureus

**Figure 1:**
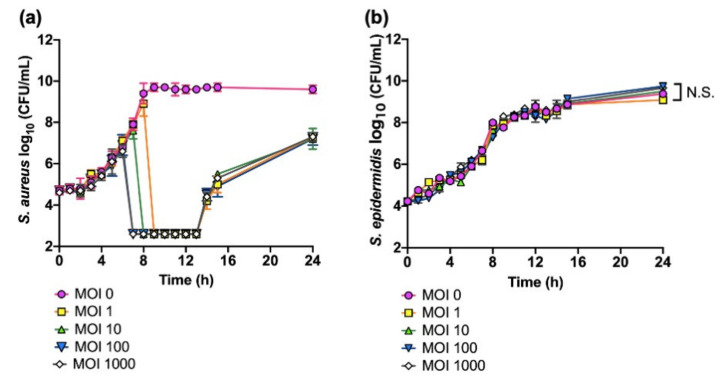
Killing assays of *S. aureus* SA-1 and *S. epidermidis* SE-4 treated with phage SaGU1. (**a**) *S. aureus* SA-1 lysis with phage SaGU1 infection. (**b**) *S. epidermidis* SE-4 growth in the presence of phage SaGU1. The culture was started with ~10^4^ CFU/mL of staphylococci and appropriate amounts of phage SaGU1. The data are presented as the mean ± SD of at least three independent experiments. The detection limit was 4.0 × 10^2^ CFU/mL. Student’s *t*-test was used to determine a significant difference between the presence and absence of phage SaGU1. N.S.; not significant.

We first investigated the effects of phage SaGU1 on the growth of *S. aureus* and non-permissive host *S. epidermidis* (Figure 1). When *S. aureus* SA-1 was infected with phage SaGU1 at an MOI of 1, cell lysis started after 8 h, and levels of SA-1 fell to below detectable levels of 400 CFU/mL between 9 h and 13 h (Figure 1a). However, the growth of SA-1 gradually started to increase after 14–24 h, suggesting the appearance of SaGU1-resistant SA-1. Increasing the relative MOI of phages shortened the time from onset to lysis and apparent elimination of SA-1 but did not affect the appearance of SaGU1 resistance. These data indicate that phage-resistant bacteria emerged at a constant rate, independent of phage concentration, and the emergence of resistant bacteria could not be prevented even if the MOI was increased, while uninfected *S. aureus* cells at the initial infection decreased with increasing phage concentration (Figure 1a). We also examined the effect of SaGU1 on the growth of *S. epidermidis*, one of the most typical beneficial bacteria. The presence of phage SaGU1 did not affect the growth of *S. epidermidis* SE-4 even in the presence of an excess number of phages (Figure 1b).

**Figure 2:**
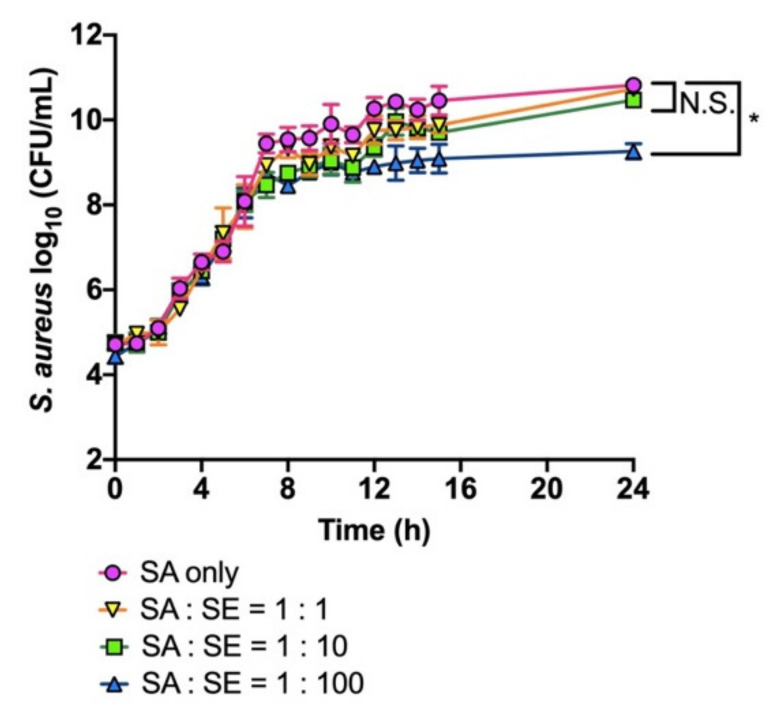
Growth of *S. aureus* SA-1 co-cultured with *S. epidermidis* SE-4. The culture was started with ~10^4^ CFU/mL of *S. aureus* SA-1 and appropriate amounts of *S. epidermidis* SE-4. The number of *S. aureus* SA-1 was determined by colony counting on LB plates with LVFX. The data are presented as the mean ± SD of at least three independent experiments. The detection limit was 4.0 × 10^2^ CFU/mL. Student’s *t*-test was used to determine a significant difference between the absence and presence of *S. epidermidis* SE-4 (*, *p* < 0.05). N.S.; not significant.

Next, we examined whether *S. epidermidis* SE-4 would affect the growth of *S. aureus* SA-1 under liquid co-culture conditions. As shown in Figure 2, *S. aureus* SA-1 initially expanded normally up to the log phase, independently of *S. epidermidis* SE-4 density. However, after 7 h, the growth of *S. aureus* SA-1 was attenuated when co-cultured with a 100 times higher dose of *S. epidermidis* SE-4, whereas a 1–10 times higher dose of *S. epidermidis* did not influence the growth of *S. aureus* SA-1 (Figure 2).

**Figure 3:**
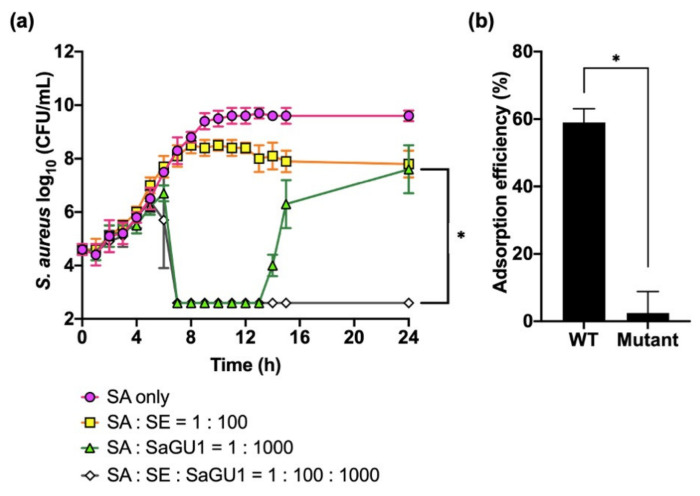
(**a**) The effect of phage SaGU1 on the growth of *S. aureus* SA-1 co-cultured with *S. epidermidis* SE-4. The cultures were started with ~10^4^ CFU/mL of *S. aureus* SA-1 and ~10^6^ CFU/mL of *S. epidermidis* SE-4 and appropriate amounts of phage SaGU1. The number of cells was determined by colony counting on LB plates with LVFX. The data are presented as the mean ± SD of at least three independent experiments. The detection limit was 4.0 × 10^2^ CFU/mL. (**b**) Adsorption assay. The adsorption efficiencies of SaGU1 for *S. aureus* SA-1 (WT) and *S. aureus* recovered from the culture of “SA: SaGU1 = 1:1000” (Mutant) after 24 h are shown. The data are presented as the mean of three independent experiments, and the error bars represent the SD. Student’s *t*-test was used to determine significance. (*, *p* < 0.001).

We finally tested the synergistic effect of phage SaGU1 and *S. epidermidis* on the growth of *S. aureus* (Figure 3a). As shown in Figure 1, SaGU1 killed *S. aureus* after 7 h, and the growth of *S. aureus* started to be restored after 14 h. However, when *S. aureus* was grown in the presence of both SaGU1 and *S. epidermidis,* unexpectedly, *S. aureus* was not restored after 14 h or up to 24 h. To check the phage susceptibility of restored bacteria, we isolated potential mutant *S. aureus* from bacterial cultures treated with SaGU1 or the one treated with SaGU1 and *S. epidermidis*. We isolated *S. aureus* from both samples (one colony from 1 mL of SaGU1- and *S. epidermidis*-treated culture) and checked the EOP of these isolates. The EOPs of isolates from SaGU1-treated culture and from SaGU1 and *S. epidermidis*-treated culture were <10^−7^ and 0.8, respectively, meaning that the isolate from SaGU1-treated culture was SaGU1 resistant. Then, we measured the adsorption efficiency of SaGU1 against the isolate (“Mutant” in Figure 3b). We found that SaGU1 could not adsorb the mutant efficiently, suggesting that the reduced EOP is due to an adsorption defect. In other words, the isolate had become resistant by blocking the adsorption of SaGU1.

These findings indicate that *S. epidermidis* SE-4 has the ability to attenuate the emergence of SaGU1-resistant *S. aureus*, in addition to suppressing the growth of *S. aureus*.

### 3.2. Effect of Phage SaGU1 in Eliminating S. aureus and on Improving Atopic Symptoms

**Figure 4:**
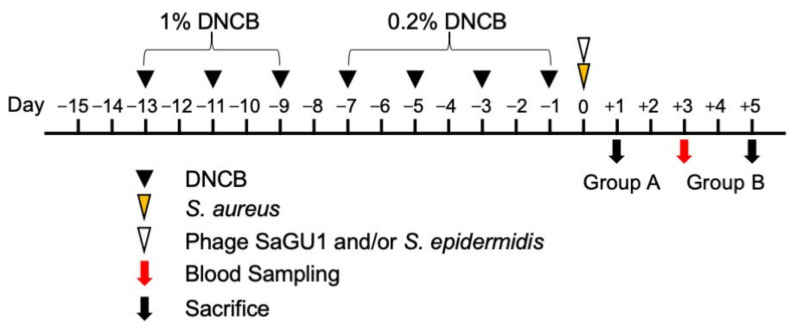
Schematic representation of the animal test for our phage therapy for atopic dermatitis (AD). Black arrowheads show the time at which 100 µL of 1% DNCB and 0.2% DNCB were applied to the back skin for sensitization. Yellow and white arrowheads show 10^5^ CFU of *S. aureus* SA-1 painting and 10^7^ CFU of *S. epidermidis* SE-4 and 10^8^ PFU SaGU1 (MOI = 1000) administration, respectively. The red arrow shows the time of blood sampling. Black arrows show time of sacrifice. Group A was used to excise the skin fragments for bacterial sampling. Group B was used to prepare the skin sections and obtain blood samples.

In order to control the growth of *S. aureus*, which exacerbates AD, we applied phage SaGU1 on atopic skin. Mice were sensitized by applying 1% DNCB to their back skin from day −13 to day −9 every other day, and 0.2% DNCB on day −7 to day −1 every other day. Typical severe skin barrier damage, such as decreased hydration, was observed in these DNCB-painted mice. On day 0, *S. aureus* SA-1, *S. epidermidis* SE-4, and/or phage SaGU1 were painted on the animals’ shaved back skin (Figure 4, see Materials and Methods).

**Figure 5:**
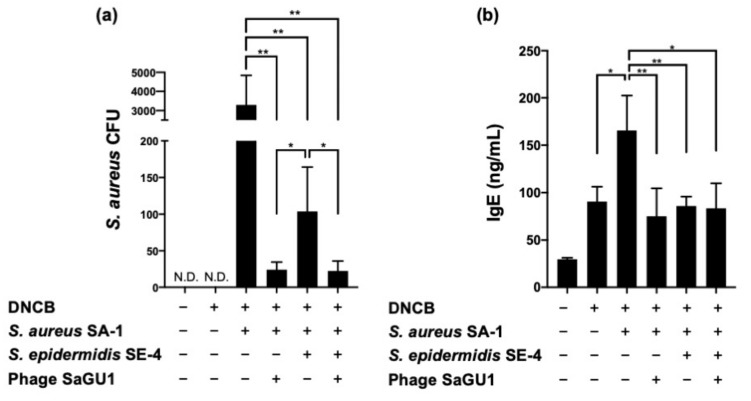
The effect of phage SaGU1 administration in DNCB-challenged mice. (**a**) The number of *S. aureus* SA-1 was determined by colony counting on LB plates with LVFX. The skin fragments were collected on day 1 after the administration of the treatment. N.D.; not detected. (**b**) The plasma IgE concentration was measured by ELISA. The blood samples were collected on day 3 after the administration of the treatment. The data are presented as the mean ± SD of at least three independent experiments. Student’s *t*-test was employed to determine a significant difference with or without SaGU1 and/or *S. epidermidis* SE-4 administration (*, *p* < 0.05 and **, *p* < 0.01).

On day 1, mice in group A were sacrificed, and their back skin was excised to isolate the bacteria (Figure 5a). Since mice were bred under SPF conditions, no bacteria were recovered from the backs of atopic mice that were not painted with *S. aureus*. For the mice that were painted only with *S. aureus*, approximately 3000 CFU of *S. aureus* were observed on the LB plate with LVFX. In contrast, the number of observed *S. aureus* was significantly decreased (to approximately 24 CFU) in mice in which *S. aureus* was suppressed by loading phage SaGU1 at MOI = 1000 (Figure 5a). We concluded that phage SaGU1 successfully eliminated *S. aureus* SA-1 not only in liquid culture conditions but also in the back skin of mice. *S. aureus* counts were also reduced by applying *S. epidermidis* SE-4 at a dose 100 times that of *S. aureus* SA-1 and by loading phage SaGU1. The decrease in *S. aureus* with the addition of *S. epidermidis* SE-4 was consistent with the results of the liquid culture (Figure 2). When both phage SaGU1 and *S. epidermidis* SE-4 were painted after *S. aureus* SA-1 was applied to the back of the mice, approximately 22 CFU of *S. aureus* were recovered. Although the recovery of *S. aureus* was lower than that of the phage alone, the difference was not statistically significant. Interestingly, all isolates recovered from the dorsal skin of mice were phage sensitive. These results indicate that SaGU1 was sufficiently more effective on mouse skin than in vitro, and the recovered phage-sensitive isolates were expected to be wild-type SA-1, which had not been exposed to SaGU1. Thus, we concluded that the combination treatment with phage SaGU1 and *S. epidermidis* SA-1 did not show a synergistic effect, at least under this condition.

On day 3, mice sensitized with DNCB showed an approximately three-fold increase in IgE concentration in their blood compared with the control mice. Moreover, the concentration of IgE increased further when the mouse backs were painted with *S. aureus* SA-1 (Figure 5b). It has been reported that various toxins produced by *S. aureus* induce IgE expression [15,16]. Treatment with phage SaGU1 significantly decreased the plasma levels of IgE by 53% in *S. aureus*-painted mice. From the viewpoint of IgE concentration, the increase caused by *S. aureus* was countered by the action of the phage, and the IgE value was restored, as in the case of DNCB treatment alone. An inhibitory effect of *S. epidermidis* SE-4 on IgE levels was also observed, similar to that in the case of phage SaGU1 treatment. It is likely that *S. epidermidis* reduced IgE levels indirectly by suppressing the growth of *S. aureus.* Since SaGU1 and *S. epidermidis* were each sufficiently effective to decrease IgE levels, it is likely that no synergistic effect was observed between them under this condition (Figure 5b). We concluded that the *S. aureus*-mediated exacerbation of AD was suppressed by phage SaGU1 or *S. epidermidis*.

### 3.3. Improvement of Atopic Dermatitis Symptoms by Phage Therapy

**Figure 6:**
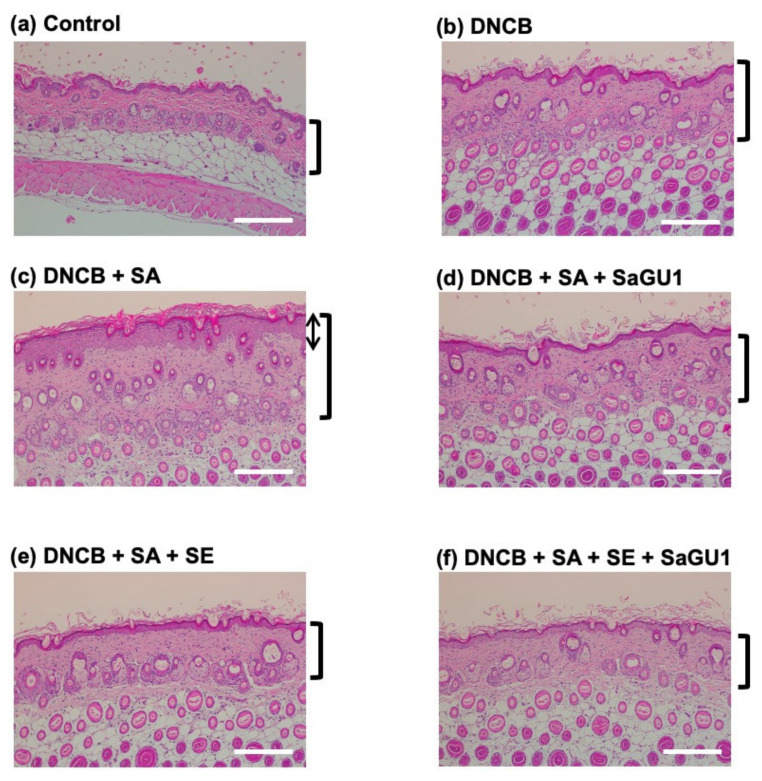
Histopathological features of skin lesions of group B mice. AD pathology was induced by DNCB in back skin specimens, and then, staphylococci and phage were applied. Tissues were excised on day 5. The sections were stained with hematoxylin-eosin. (**a**) Control, no DNCB sensitization and no *S. aureus* SA-1 painting; (**b**) with DNCB sensitization and no *S. aureus* SA-1 painting; (**c**) with DNCB sensitization and *S. aureus* SA-1 painting; (**d**) with DNCB sensitization, *S. aureus* SA-1 painting, and SaGU1 administration; (**e**) with DNCB sensitization, *S. aureus* SA-1 painting, and *S. epidermidis* SE-4 administration; (**f**) with DNCB sensitization, *S. aureus* SA-1 painting, and administration of both *S. epidermidis* SE-4 and SaGU1. Scale bars correspond to 200 µm. Square brackets on the right indicate epidermis thickness, and the arrow in (**c**) indicates the thickness of the stratum corneum.

The histopathological features of the back skin from DNCB-painted atopic mice are shown in Figure 6. Epidermal thickening in the dermis was observed (Figure 6b). The pathological increase in epidermal thickness was further enhanced by *S. aureus* SA-1 painting (Figure 6c). The dramatic increase in epidermal thickness of *S. aureus* SA-1-painted mice was significantly attenuated by phage SaGU1 treatment (Figure 6d). *S. epidermidis* SE-4 also inhibited DNCB-induced epidermal hyperplasia in AD (Figure 6e). Moreover, Figure 6f seems to indicate a potential additive effect of *S. epidermidis* and SaGU1. These skin pathological findings correlated with changes in IgE values (Figure 5b), and skin thickening was suppressed as the IgE level decreased. In this phage therapy study, we were not able to observe complete restoration of the skin; however, since the amelioration of atopic symptoms correlated with plasma IgE values and histological analysis of skin sections, we concluded that the administration of phage SaGU1 and *S. epidermidis* SE-4 was an effective AD treatment.

## 4. Discussion

Phage therapy is a potential treatment for bacterial infectious disease, calling for the exploration of host specificity of phages for the targeted killing of pathogenic bacteria without disturbing natural microbiota. Although phage therapy is promising, there are chances of the emergence of phage-resistant bacteria. Therefore, researchers are working on combination therapies using phages and other treatments, e.g., antibiotics [17]. This can be effective in critical situations or in situations where wiping out the bacteria does not perturb natural microbiota, e.g., bacteremia and lung infectious diseases. In other words, combination therapy using antibiotics is not suitable for environments where health is affected by resident microbiota, such as the intestines, oral cavity, skin, etc., as microbiota is a key factor in the maintenance of the health of these tissues, and therefore, disturbing this microbiota can induce and exacerbate various diseases [18]. There is another way to use probiotics as a therapy without disturbing the microbiota; for instance, a study on the clinical use of probiotics is being conducted for the recovery of dysbiosis [19]. It has been reported that administration of beneficial bacteria such as *Lactobacillus* in the intestinal microbiota helps to cure antibiotic-associated diarrhea, bacterial vaginosis, *Helicobacter pylori* infection, etc. [20,21,22]. Improvement of the intestinal microbiota may relieve allergic symptoms. There are reports that probiotics are effective in treating AD [23].

In the present study, phage therapy in combination with *S. epidermidis,* a beneficial symbiotic bacterium, was used to treat atopic dermatitis. *S. epidermidis* has been reported to work as a skin barrier and also have an antibacterial effect against *S. aureus* [24,25,26]. We assume that the inhibitory effect of *S. epidermidis* on *S. aureus* growth is due to such antimicrobial activity or simply because of nutrient depletion. In our in vivo experiment, no synergistic effect was observed in the combined use of phage SaGU1 and *S. epidermidis*. This might be ascribed to the optimum killing of *S. aureus* by phage SaGU1 or *S. epidermidis.* To confirm the combinatorial effect, in vivo tests under different conditions such as treatment for more severely ill mice and long-term follow-up are required in future. Meanwhile, we observed that the combination of phage SaGU1 and *S. epidermidis* suppressed the growth of phage-resistant *S. aureus* in vitro. Therefore, we suggest here that the combined use of phage and beneficial symbiotic bacteria may be useful for the treatment of skin bacterial infections.

Our phage therapy experiment was performed under controlled conditions, and 1 h separation between the application of bacteria and the subsequent application of a 1000 times higher number of phages may be unlikely to represent a realistic model of infection or a realistic mode of treatment. Such a high MOI may well result in passive lysis of the administered bacteria. However, the exact number of bacteria in the affected area cannot be known in an actual treatment [27]. Further, due to inconsistent access between phages and the host bacteria, it is difficult to determine the dosage of phage by MOI.

Recent research using mouse skin infection models has revealed that phage therapy is effective to treat skin infections caused by *Pseudomonas aeruginosa*, *Klebsiella pneumoniae*, and *Mycobacterium ulcerans* [8,28,29]. The administration of phages along with beneficial bacteria for skin infections protects the microbiota and also has an advantage of its direct administration to the affected area. Our results indicate that phages and *S. epidermidis* may be useful to treat atopic dermatitis and skin infection. Although the combinatorial use of probiotics and phages targeting *S. aureus* has been reported [30,31], to our knowledge, this is the first report of combined use for a potential treatment of *S. aureus*-associated skin infection. In this study, we used only a specific phage, SaGU1, in all experiments; nevertheless, we believe the results obtained here will be useful for other phage therapy research. This study presents a new antimicrobial therapy involving the simultaneous administration of phages and probiotics, and we believe that it will have sufficient impact in the field of microbiology. Further reports on the combinatorial approach for other infectious diseases are expected in future.
